# Supplementary material for: Growth-inhibiting effects of the unconventional plant APYRASE 7 of Arabidopsis thaliana influences the LRX/RALF/FER growth regulatory module
Source: PLoS Genet. 2024 Jan 8;20(1):e1011087. doi: 10.1371/journal.pgen.1011087 (PMC10824444; doi:10.1371/journal.pgen.1011087)
Supplement: S1 Fig — (A) List of SNPs in coding sequences around ROL16 (At4g19180, indicated in bold), as obtained from WGS. (B) The C to T mutation in ROL16 was confirmed by sequencing. As the coding strand orientation is opposite of the WGS data, it is a G to A polymorphism (indicated with black arrow). A CAPS marker (for details, see Material and Methods) was established for simple detection. (C) Upper panel; schematic drawing of the APY7 protein and genomic DNA, with the protein coding sequence (indicated in grey) interrupted by an intron. qRT-PCR was performed with two primer pairs, located in the 5’ and 3’ UTRs, respectively. Total RNA was extracted from wild type (Col), lrx1, lrx1 rol16, and lrx1 apy7-1 mutant seedlings. The lower panel shows quantification of RNA levels with the wild type set to 1. (D) Comparison of APY7 with APY1,2,6 of Arabidopsis and a human NTPDase. APY6 is the only Arabidopsis APY, besides APY7, to have two TMDs. APYs have five conserved motifs (apyrase conserved regions, ACRs), in APY7 corresponding to positions 150–157 (ACR1), 236–246 (ACR2), 281–293 (ACR3), 312–319 (ACR4), and 566–569 (ACR5). The amino acid sequences of the ACRs are listed. APY7 has a C-terminal extension not found in any other APY protein in Arabidopsis, the biological significance of which remains elusive. (DOCX) [file pgen.1011087.s001.docx]

**A**

| **position** | **gene identifier** | **mutation** | **coverage** |
| --- | --- | --- | --- |
| 9032480 | AT4G15920 | -A | 23/24 |
| 9051307 | AT4G15970 | T- | 34/34 |
| 9138630 | AT4G16144 | -T | 25/26 |
| 9259187 | AT4G16390 | TG | 21/21 |
| 9288701 | AT4G16470 | -G | 73/75 |
| **10486589** | **AT4G19180** | **CT** | **80/80** |
| 16280059 | AT4G33970 | GT | 21/22 |
| 16807916 | AT4G35335 | -G | 85/87 |
| 17371721 | AT4G36870 | -C | 51/53 |

**B**


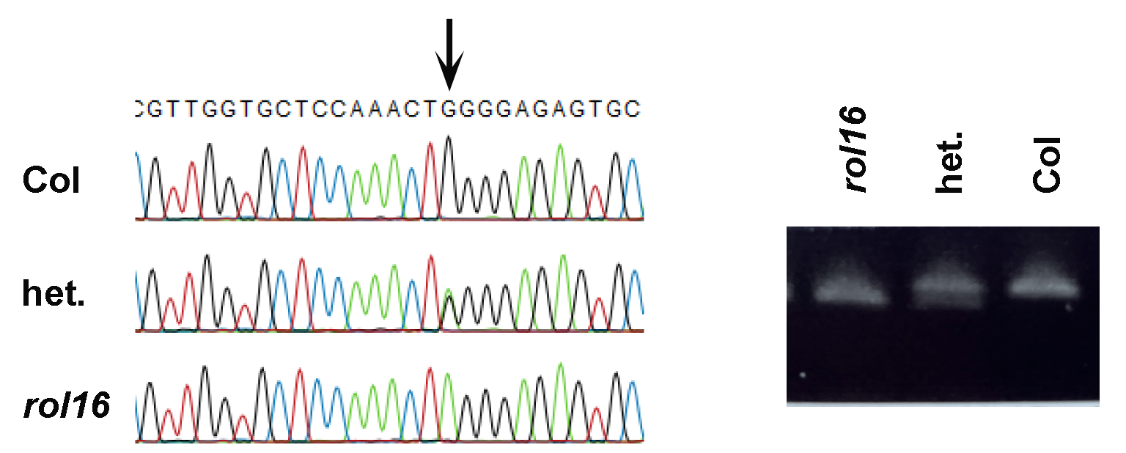


**C**

**
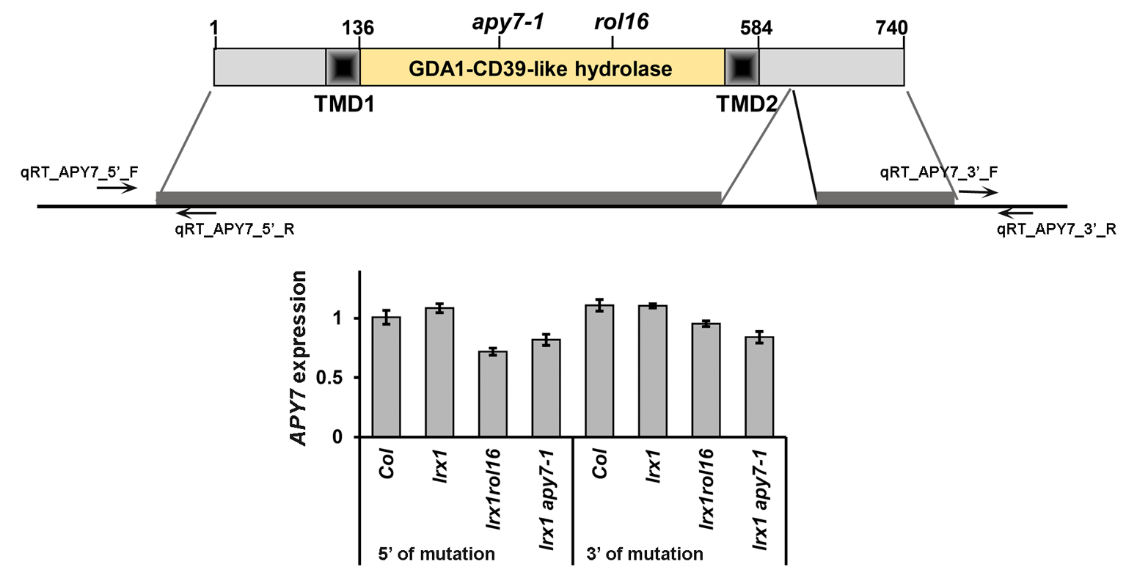
**

**D**

**
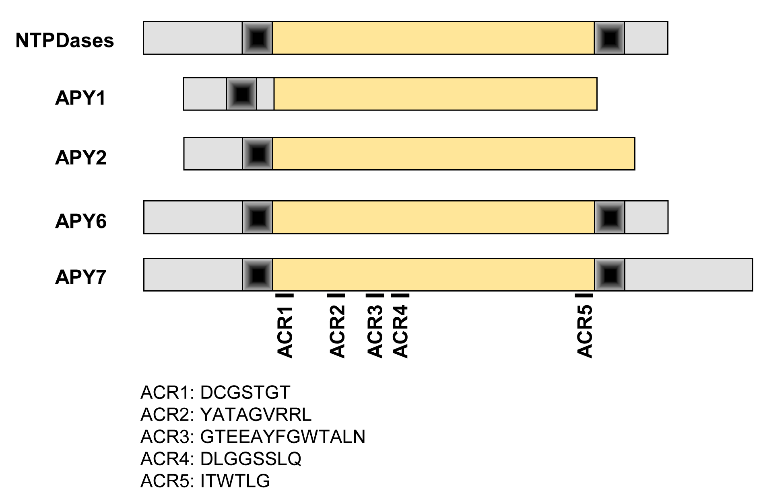
**

**Suppl. Figure S1** Mutation in *APY7* and comparison to related APYs.

**(A)** List of SNPs in coding sequences around *ROL16* (At4g19180, indicated in bold), as obtained from WGS. **(B)** The C to T mutation in *ROL16* was confirmed by sequencing. As the coding strand orientation is opposite of the WGS data, it is a G to A polymorphism (indicated with black arrow). A CAPS marker (for details, see Material and Methods) was established for simple detection. **(C)** Upper panel; schematic drawing of the APY7 protein and genomic DNA, with the protein coding sequence (indicated in grey) interrupted by an intron. qRT-PCR was performed with two primer pairs, located in the 5’ and 3’ UTRs, respectively. Total RNA was extracted from wild type (Col), *lrx1,* *lrx1 rol16*, and *lrx1 apy7-1* mutant seedlings. The lower panel shows quantification of RNA levels with the wild type set to 1. **(D)** Comparison of APY7 with APY1,2,6 of Arabidopsis and a human NTPDase. APY6 is the only Arabidopsis APY, besides APY7, to have two TMDs. APYs have five conserved motifs (apyrase conserved regions, ACRs), in APY7 corresponding to positions 150-157 (ACR1), 236-246 (ACR2), 281-293 (ACR3), 312-319 (ACR4), and 566-569 (ACR5). The amino acid sequences of the ACRs are listed. APY7 has a C-terminal extension not found in any other APY protein in Arabidopsis, the biological significance of which remains elusive.
