# Supplementary material for: Growth-inhibiting effects of the unconventional plant APYRASE 7 of Arabidopsis thaliana influences the LRX/RALF/FER growth regulatory module
Source: PLoS Genet. 2024 Jan 8;20(1):e1011087. doi: 10.1371/journal.pgen.1011087 (PMC10824444; doi:10.1371/journal.pgen.1011087)
Supplement: S2 Fig — (A-D) APY7::GUS fusion construct transformed into Col-0 for expression analysis. The 6-days-old seedlings were stained with GUS for 3–4 hours and the reaction was stopped by adding 70% ethanol. APY7::GUS activity was observed in the root tissue. GUS staining was observed at the lateral root initiation site (A), in the vasculature of the maturation/ differentiation zone (B), diffused expression in the elongation or cell division zone (C), and high expression in the root tip (D). (E) GFP fluorescence induced by an APY7::APY7-GFP construct reveals expression in all cells with a punctate structure likely representing the Golgi. Bar = 500 μm (A-D have the same magnification). (DOCX) [file pgen.1011087.s002.docx]

**
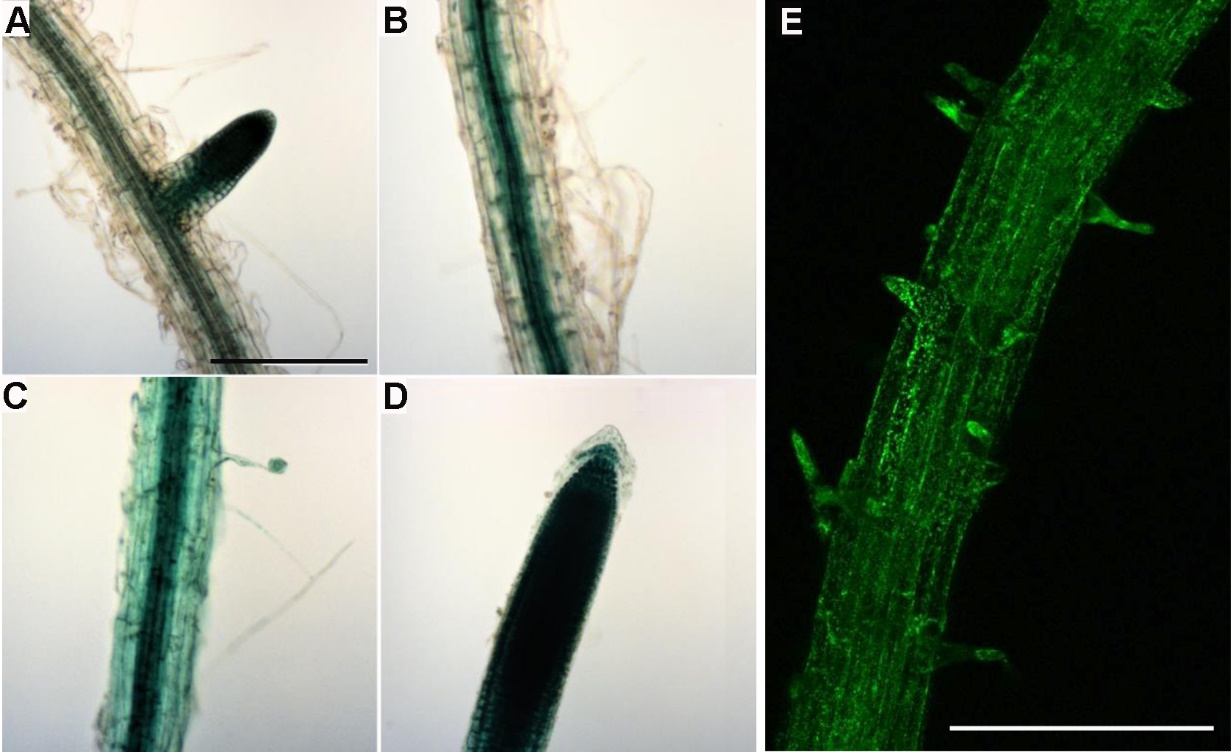
**

**Suppl. Figure S2** Expression of *APY7* in roots of *Arabidopsis thaliana*.

**(A-D)** *APY7::GUS* fusion construct transformed into Col-0 for expression analysis. The 6-days-old seedlings were stained with GUS for 3-4 hours and the reaction was stopped by adding 70 % ethanol. *APY7::GUS* activity was observed in the root tissue. GUS staining was observed at the lateral root initiation site **(A)**, in the vasculature of the maturation/ differentiation zone **(B)**, diffused expression in the elongation or cell division zone **(C)**, and high expression in the root tip **(D)**. **(E)** GFP fluorescence induced by an *APY7::APY7-GFP* construct reveals expression in all cells with a punctate structure likely representing the Golgi. Bar = 500 µm (A-D have the same magnification).
