# Supplementary material for: Growth-inhibiting effects of the unconventional plant APYRASE 7 of Arabidopsis thaliana influences the LRX/RALF/FER growth regulatory module
Source: PLoS Genet. 2024 Jan 8;20(1):e1011087. doi: 10.1371/journal.pgen.1011087 (PMC10824444; doi:10.1371/journal.pgen.1011087)
Supplement: S3 Fig — (A) rol16 alleviates the root hair developmental defect induced by the fer-4 knock-out mutant, with more root hairs successfully entering the elongation phase, while remaining shorter than in the wild type. Bar = 300 μm. Classification of different types of root hair defects in the wild type (Col) compared to fer-4 and fer-4 rol16. Different letters indicate significant differences between the lines (student’s t-test, n>250, p<0.001) (B) The frequency of homozygous mutant fer-4 is strongly reduced by five-fold in the rol16 mutant background compared to a wild-type ROL16 background, suggesting a genetic interaction between rol16 and fer-4. (C) Siliques of fer-4 rol16 double mutants are shorter and contain less seed than the respective single mutants. Bar = 1cm. (DOCX) [file pgen.1011087.s003.docx]

**A**

**
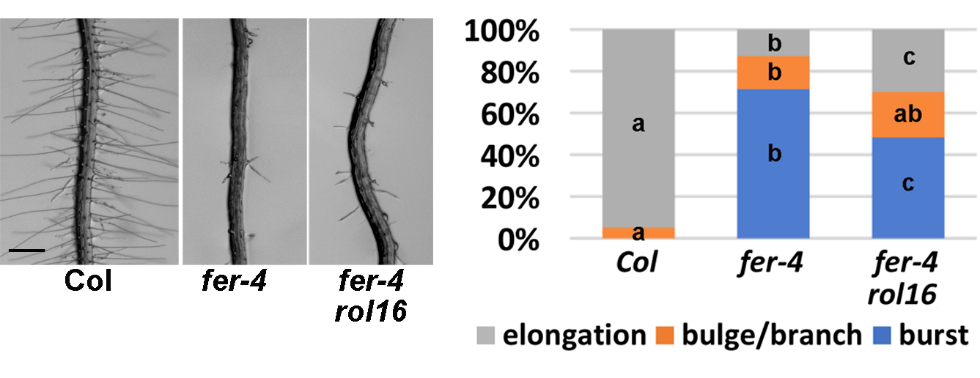
**

**B**

| parental genotype | total number of  progeny analyzed | number of homoz. mutant progeny | frequency of homoz. mutant progeny [%] |
| --- | --- | --- | --- |
| *fer-4* ^+/-^ | 800 | 60 | 7.5 |
| *rol16* ^+/-^ | 840 | 190 | 22 |
| *rol16* ^-/-^; *fer-4* ^+/-^ | 800 | 12 | 1.5 |

**C**


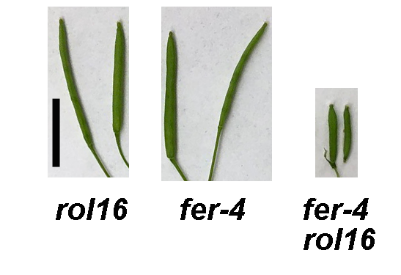


**Suppl. Figure S3** Interaction of the *rol16* and *fer* mutations.

**(A)** *rol16* alleviates the root hair developmental defect induced by the *fer-4* knock-out mutant, with more root hairs successfully entering the elongation phase, while remaining shorter than in the wild type. Bar =300 µm. Classification of different types of root hair defects in the wild type (Col) compared to *fer-4* and *fer-4 rol16*. Different letters indicate significant differences between the lines (student’s t-test, n>250, p<0.001) **(B)** The frequency of homozygous mutant *fer-4* is strongly reduced by five-fold in the *rol16* mutant background compared to a wild-type *ROL16* background, suggesting a genetic interaction between *rol16* and *fer-4*. **(C)** Siliques of *fer-4 rol16* double mutants are shorter and contain less seed than the respective single mutants. Bar= 1cm.
