# Supplementary material for: Growth-inhibiting effects of the unconventional plant APYRASE 7 of Arabidopsis thaliana influences the LRX/RALF/FER growth regulatory module
Source: PLoS Genet. 2024 Jan 8;20(1):e1011087. doi: 10.1371/journal.pgen.1011087 (PMC10824444; doi:10.1371/journal.pgen.1011087)
Supplement: S4 Fig — Seedlings and stem sections were incubated in Wiesner staining solution. (A) Seedling hypocotyls, (B) seedling roots, (C) stem sections. Both distribution and intensity of lignification are comparable between the wild type and the rol16 mutant plants. Bars = 300 μm (A), 200 μm (B), 1 mm (C). (DOCX) [file pgen.1011087.s004.docx]

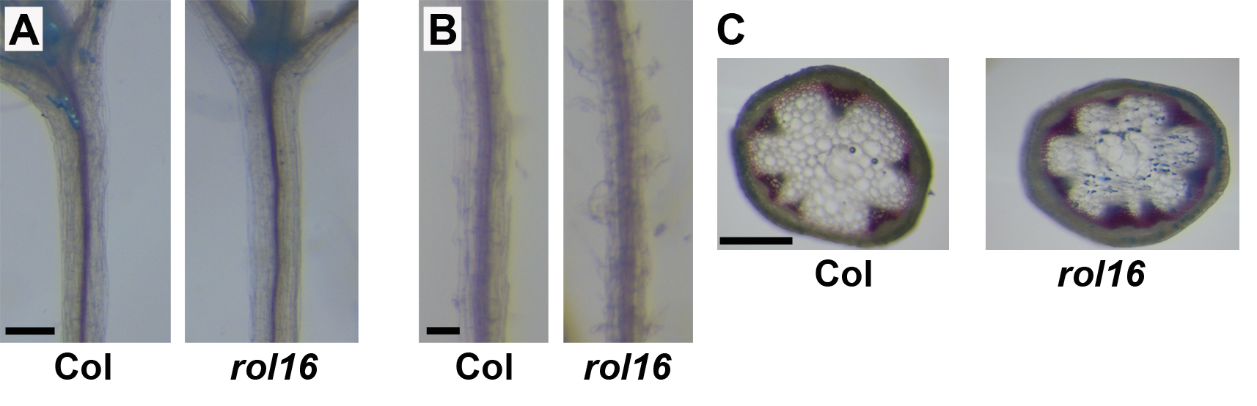


**Suppl. Figure S4** Comparable lignification of wild type and *rol16***.**

Seedlings and stem sections were incubated in Wiesner staining solution. **(A)** Seedling hypocotyls, **(B)** seedling roots, **(C)** stem sections. Both distribution and intensity of lignification are comparable between the wild type and the *rol16* mutant plants. Bars=300 μm (A), 200 μm (B), 1 mm (C).
