# Supplementary material for: Growth-inhibiting effects of the unconventional plant APYRASE 7 of Arabidopsis thaliana influences the LRX/RALF/FER growth regulatory module
Source: PLoS Genet. 2024 Jan 8;20(1):e1011087. doi: 10.1371/journal.pgen.1011087 (PMC10824444; doi:10.1371/journal.pgen.1011087)
Supplement: S5 Fig — Schematic representation on the left: the ratio of the total length of the seedlings root (L) over the progression on the Y-axis (ΔY) describe the arccosinus of Θ, a mathematical description of growth behaviour [74]. Stronger root scewing results in an increase in angle Θ. Right: example of Col versus rol16 mutant seedlings grown with 0 mM ATP (top) and 1 mM ATP (bottom), used for data acquisition. It reveals that with eATP treatment, Θ values are bigger in rol16 mutants compared ot the wild type. (DOCX) [file pgen.1011087.s005.docx]

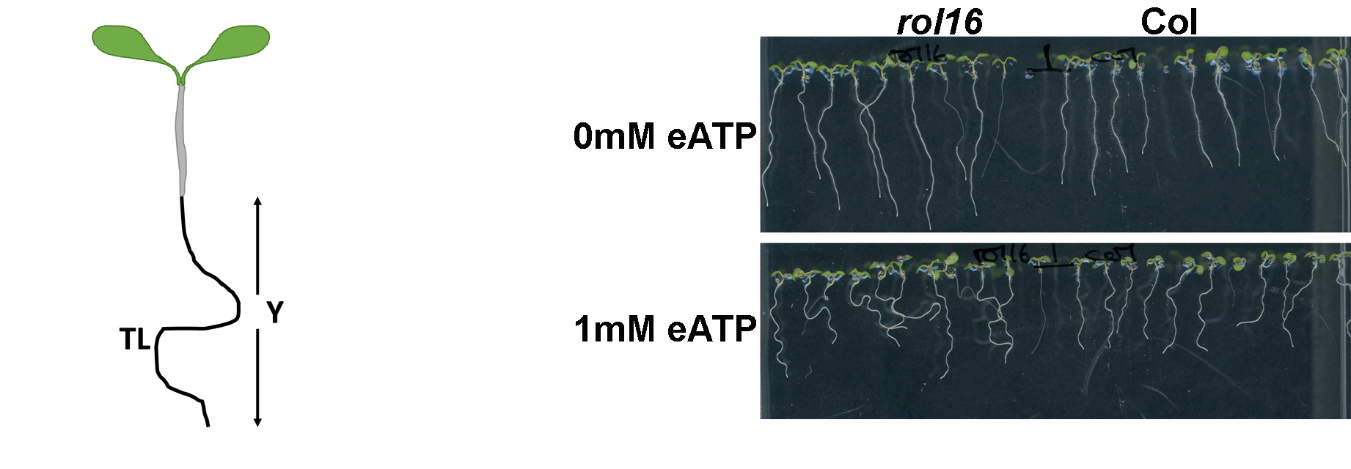


**Suppl. Figure S5** Quantification of root skewing.

Schematic representation on the left: the ratio of the total length of the seedlings root (L) over the progression on the Y-axis (ΔY) describe the arccosinus of Θ, a mathematical description of growth behaviour [74]. Stronger root scewing results in an increase in angle Θ. Right: example of Col versus *rol16* mutant seedlings grown with 0 mM ATP (top) and 1 mM ATP (bottom), used for data acquisition. It reveals that with eATP treatment, Θ values are bigger in *rol16* mutants compared ot the wild type.
