# Supplementary material for: Growth-inhibiting effects of the unconventional plant APYRASE 7 of Arabidopsis thaliana influences the LRX/RALF/FER growth regulatory module
Source: PLoS Genet. 2024 Jan 8;20(1):e1011087. doi: 10.1371/journal.pgen.1011087 (PMC10824444; doi:10.1371/journal.pgen.1011087)
Supplement: S6 Fig — (A) P2K1/DORN1 (At5g60300) and P2K2 (At3g45430) encode lectin-like receptor kinases that bind ATP. Numbers indicate amino acid positions, the mutants being used in this work are indicated, the dorn1 mutant is the T-DNA insertion line SALK_042209 with the insertion site corresponding to amino acid codon 92, p2k2 represents CRISPR/CAS9 -induced mutations at the codon 107 that change the reading frame and terminate translation after around 30 amino acids. Numbers indicate amino acid positions. (B) P2K2 expression levels were determined by qRT-PCR on RNA samples from wild-type Col and different p2k2 alleles, with Col arbitrarily set to 1. (DOCX) [file pgen.1011087.s006.docx]

**A**





**B**

**

**

**Suppl. Figure S6** DORN1 and P2K2 protein structure.

**(A)** *P2K1*/*DORN1* (At5g60300) and *P2K2* (At3g45430) encode lectin-like receptor kinases that bind ATP. Numbers indicate amino acid positions, the mutants being used in this work are indicated, the *dorn1* mutant is the T-DNA insertion line SALK_042209 with the insertion site corresponding to amino acid codon 92, *p2k2* represents *CRISPR/CAS9* -induced mutations at the codon 107 that change the reading frame and terminate translation after around 30 amino acids. Numbers indicate amino acid positions. **(B)** *P2K2* expression levels were determined by qRT-PCR on RNA samples from wild-type Col and different *p2k2* alleles, with Col arbitrarily set to 1.
