# Supplementary material for: Growth-inhibiting effects of the unconventional plant APYRASE 7 of Arabidopsis thaliana influences the LRX/RALF/FER growth regulatory module
Source: PLoS Genet. 2024 Jan 8;20(1):e1011087. doi: 10.1371/journal.pgen.1011087 (PMC10824444; doi:10.1371/journal.pgen.1011087)
Supplement: S7 Fig — Plants expressing APY7-GFP with the plasma membrane marker LTI6b-RFP were used for colocalization. A virtual section through cells revealed strong RFP signal at the cell periphery due to LTI6b-RFP fluorescence at the plasma membrane. By contrast, no signal beyond background could be identified with the GFP filter identifying APY7-GFP. (DOCX) [file pgen.1011087.s007.docx]

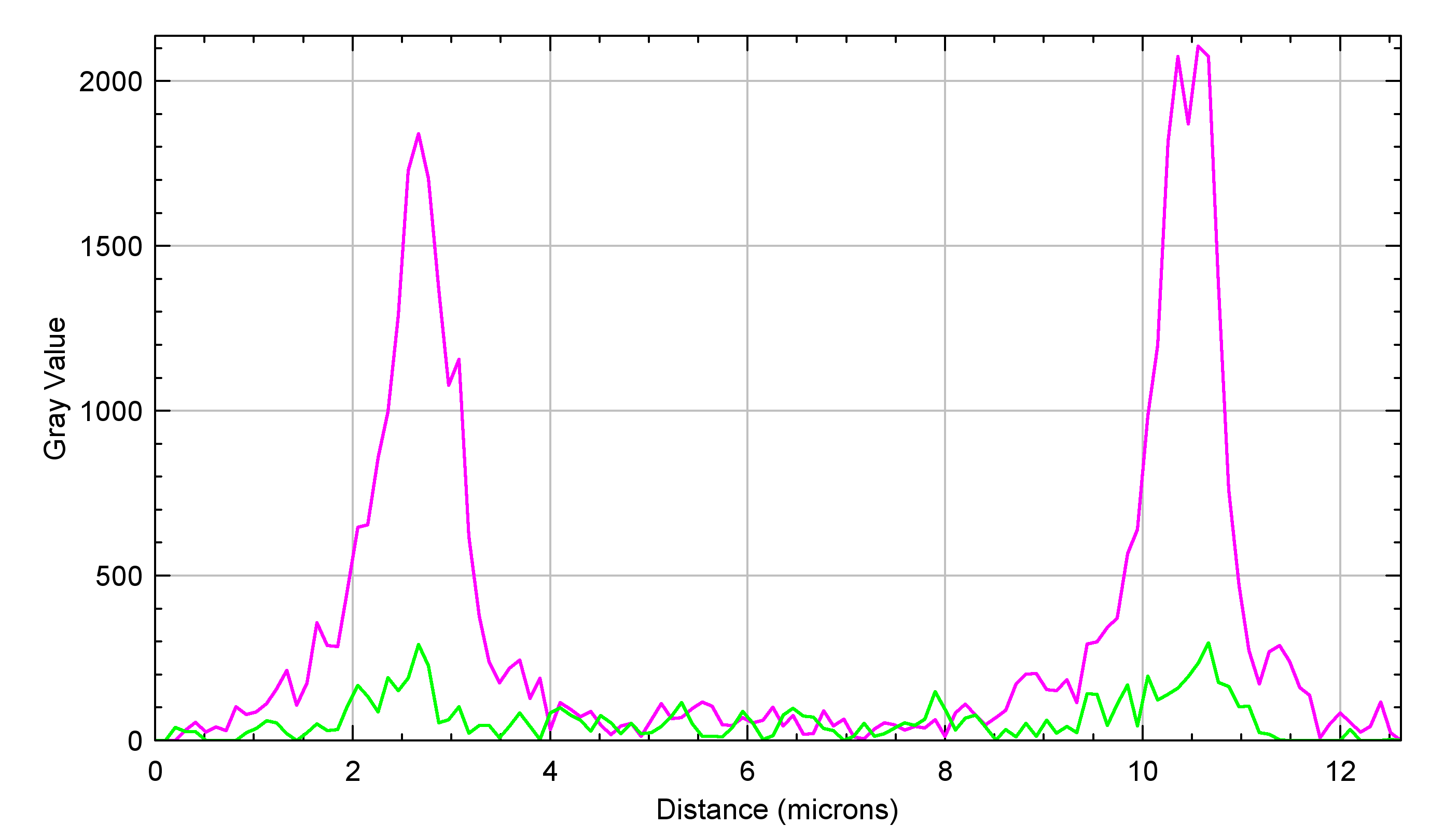


**Suppl. Figure S7** APY7 is not a genuine plasma membrane protein.

Plants expressing APY7-GFP with the plasma membrane marker LTI6b-RFP were used for colocalization. A virtual section through cells revealed strong RFP signal at the cell periphery due to LTI6b-RFP fluorescence at the plasma membrane. By contrast, no signal beyond background could be identified with the GFP filter identifying APY7-GFP.
