# Supplementary material for: Growth-inhibiting effects of the unconventional plant APYRASE 7 of Arabidopsis thaliana influences the LRX/RALF/FER growth regulatory module
Source: PLoS Genet. 2024 Jan 8;20(1):e1011087. doi: 10.1371/journal.pgen.1011087 (PMC10824444; doi:10.1371/journal.pgen.1011087)
Supplement: S1 Table — Positions in bold (SG23) indicates SNP compared to genomic DNA to create a CAPS marker with a SpeI in rol16. (DOCX) [file pgen.1011087.s008.docx]

Suppl. Table S1

| ***rol16* Primers** |  |
| --- | --- |
| SG23 | CAAGTGCGCTGCACTCTC**A**C |
| SG24 | GCGTTTGATAGGTCTGTTGTTCA |
|  |  |
| ***apy7-1* Primers** |  |
| SG75 | CTCCATGTTTTGCGTTTGACC |
| SG76 | CGTGCTTATGATCGGATGGAGA |
|  |  |
| ***APY7::APY7-GFP*** |  |
| APYProm_F | TGAACTAGTATTCTAGGACTCTATATGGTCAC |
| APYProm_R | TGAGGCGCGCCTTCTCTTGCCTACATATACTAAC |
| APY7_F | GGCGCGCCATGGTTTTTGGTAGGATCACTG |
| APY7_R | GGCGCGCCTCATTTTGAGCATGTGTG |
| GFP_AscI_F | GGCGCGCCATGAGTAAAGGAGAAGAAG |
| GFP_SacI_R | TCAGAGCTCTTAGTGGTGGTGGTGGTG |
|  |  |
| *dorn1-3* |  |
| dorn1_F | CTGAATACTTGCGTCTCCTGC |
| dorn1_R | CAGCTTGCGAGGTTATGATTC |
|  |  |
| P2K2 crispr primers |  |
| P2K2_KI1_F | ATTGGCCCGTGTGAAATCCACAGT |
| P2K2_KI1_R | AAACACTGTGGATTTCACACGGGC |
|  |  |
| qRT-PCR primers |  |
| qRT_apy7-1_5’_F | TATGCCAATGCGTGTTTCTGAAG |
| qRT_apy7-1_5’_R | TGGAACAGATGATTGTGAACCCG |
| qRT_apy7-1_3’_F | CACTTCCACACCAGCCTAATTTT |
| qRT_apy7-1_3’_R | AAGGGTAAAGAGGAAGAGAGTGA |
| P2K2_qRT_F2 | CCAGCAATGGGACAAGTGGTTCT |
| P2K2_qRT_R2 | GTGAGGCTGCATCGACAACTACA |
| EFα_F | TGAGCACGCTCTTCTTGCTTTCA |
| EFα_R | GGTGGTGGCATCCATCTTGTTACA |
| ACT2_F | CTTGCACCAAGCAGCATGAA |
| ACT2_R | CCGATCCAGACACTGTACTTCCTT |
| UBQ10_F | GGCCTTGTATAATCCCTGATGAATAAG |
| UBQ10_R | AAAGAGATAACAGGAACGGAAACATAGT |

Primers used in this study. Positions in bold (SG23) indicates SNP compared to genomic DNA to create a CAPS marker with a SpeI in *rol16*.
