# Supplementary material for: Growth-inhibiting effects of the unconventional plant APYRASE 7 of Arabidopsis thaliana influences the LRX/RALF/FER growth regulatory module
Source: PLoS Genet. 2024 Jan 8;20(1):e1011087. doi: 10.1371/journal.pgen.1011087 (PMC10824444; doi:10.1371/journal.pgen.1011087)
Supplement: S2 Table — A list of these antibodies, their specificities and list of publications describing them can be found at https://plantcellwalls.leeds.ac.uk/wp-content/uploads/sites/103/2021/11/JPKab2021.pdf (DOCX) [file pgen.1011087.s009.docx]

S2 Table

| ***mAbs*** |  |
| --- | --- |
| LM 1 | extensins /AGPs |
| LM 2 | AGP |
| LM5 | 1-4 Gal |
| LM 6 | 1-5 Ara |
| LM 7 | partially non-block wise methylesterified HG |
| LM 10 | NRE xylan |
| LM 11 | 1-4 xylosyl |
| LM 15 | xyloglucan |
| LM 18 | partially methylesterified HG |
| LM19 | unesterified HG |
| LM 20 | highly methylesterified HG |
| Jim 7 | partially methylesterified HG |
| JIM 11 | extensin |
| JIM 12 | extensin |
| JIM 13 | AGPs |
| JIM 20 | extensin |

Monoclonal antibodies used in this work. A list of these antibodies, their specificities and list of publications describing them can be found at

<https://plantcellwalls.leeds.ac.uk/wp-content/uploads/sites/103/2021/11/JPKab2021.pdf>
