# Supplementary material for: Growth-inhibiting effects of the unconventional plant APYRASE 7 of Arabidopsis thaliana influences the LRX/RALF/FER growth regulatory module
Source: PLoS Genet. 2024 Jan 8;20(1):e1011087. doi: 10.1371/journal.pgen.1011087 (PMC10824444; doi:10.1371/journal.pgen.1011087)
Supplement: Attachment — Submitted filename: response to reviewers comments_final.docx [file pgen.1011087.s010.docx]

We are grateful to both reviewers for their suggestions how to improve the manuscript.

The responses to their points raised are indicated in red.

**Reviewer #1**: The manuscript by Gupta et al. describes a relationship between APYRASE7 (APY7) and LRX1/FER/RALF module. By using genetics approaches, the authors observed that APY7 suppress the growth defects in lrx and fer mutants. Further, they found that APY7 modulates the growth-inhibiting effect of RALF1, influences the cell wall components, and alters apoplastic pH. This manuscript focused on an interesting topic. However, there are quite a few critical comments/concerns that should be adequately addressed. Especially, how APY7 spatially inhibits LRX1/FER/RALF? how to explain the cell elongate faster with a more alkali apoplastic pH in apy7-1? I suggest the authors address the following comments and significantly strengthen this work by additional data.

Major
1. APY7, LRX and FER have different subcellular localization patterns. It is hard to imagine how Golgi apparatus-localized APY7 spatially work with cell surface-localized RALF1/LRX/FER.

We agree that it is hard to conceive a situation with a direct interaction between LRX1/FER and APY7. While LRX-FER interactions have been shown by different groups, we do not want to claim an interaction of either LRX1, RALFs, or FER with APY7. We much rather think that this is a genetic interaction and that these proteins play roles in generally the same process but at different time points and cellular locations. We have modified the text to avoid confusion in this issue (the title on lane 192 was changed, and the discussion to make this point clearer (lane 453-460).

2. I am not convinced that eATP induces the gravity-dependent root skewing of rol16. Because root skewing does not mean that plant do not have gravitropism. It would be necessary to observe root growth using a soil-based method.

We acknowledge that mixing skewing and gravitropism is inaccurate and therefore refer to root skewing only (lanes 356, 362,367,372, Suppl. Figure S5 lane 88, 91). A number of publications demonstrate that eATP induces root skewing and apyrases are involved in the response to eATP (e.g. Yang et al, Plant Cell and Physiology, 2015). Hence, based on the literature, we feel that making this connection is justified.

In Suppl Fig S5, we schematically show the way the data were produced. The original schematic drawing of the seedling had to be replaced by a new drawing, due to copy-right issues. The legend was also adapted, but the content of this figure remains the same.

3. In Fig1A, APY7::APY7-GFP is complemented into the lrx rol16 double mutant background. It would be better to select at least two lines to observe the root hair phenotype. Besides, can different abundances of APY7 restore the defect in root hair development at different levels?

A more detailed analysis of the complementation efficiency of transgenic lines accumulating distinct levels of APY7-GFP (Fig1 C) are now shown in the new panel Fig 1D. The text was modified accordingly (lanes 178-184). The data points were obtained from 5 or more seedlings per line to make this a statistically robust data set.

4. An additional experiment of APY7::APY-GFP in fer-4 apy7-1 should be added in Fig 3C to demonstrate that APY7 has a function in growth processes that are influenced by RALF1.

We are not sure we understand this point. To complement a *rol16 fer-4* mutant with APY7-GPF would still result in a RALF1-insensitiv line due to the *fer-4* mutation. Also, obtaining a *rol16 fer-4* APY-GFP line has proven difficult due to the poor fertility of the *rol16 fer-4* double mutant. After many attempts, we are now at the F1 generation of a cross of APY7-GFP T2-4 *x rol16 fer-4*, we would need another 3-4 months to complete the experiment.

5. The peak map or raw data of GWS and PCR gel images of SNP molecular markers should be provided in supplementary data.

New S1A Fig and S1B Fig panels were introduced to show these data. The legend of S1 Fig was changed accordingly.

6. Except for genetics, biochemical data are needed to support the conclusion that APY7 inhibits the LRX/RALF/FER pathway, such as interactions-related experiments using BiFC and Co-IP.

As mentioned above, we do not claim a direct interaction of FER/LRX with APY7. In fact, we believe that they do not interact, yet function at different locations and steps of the same/related process. We have changed the discussion accordingly to make this point clearer (see response to point Nr 1).

7. Further experiment could be carried out to characterize the APY7-dependent changes in structures and functions of the cell wall. For instance, chemistry approaches can be used to quantify the content and composition of cellulose, hemicellulose, and lignin in apy 7-1 mutant, electron microscopy to observe the ultrastructure and morphology of its cell wall.

We have now included a quantification of monosaccharides of cell wall fractions, demonstrating that pectin and hemicelluloses (including xylan) are reduced in the *rol16* mutant (new panel Fig 5F). We are grateful to the reviewer suggesting this experiment, because the outcome suggests that golgi-derived cell wall polysaccharides are affected in *rol16*. This hypothesis is mentioned in the discussion (Lane 492-498). Analysis of lignin revealed no altered accumulation of lignin in *rol16* seedlings, nor a change in lignin distribution or abundance in inflorescent stems of *rol16* compared to the wild type plants. The data are shown in the new S4 Fig, corresponding text in the Result (lane 309-311) and Material and Methods (lanes 618-621) and discussion (lanes 498-501).

An analysis of cell wall ultrastructures by electron microscopy might possibly be informative, but this would be almost a project on its own. We therefore chose to focus on the above-mentioned monosaccharide analysis.

8. The phenotype of root hairs in the picture needs corresponding index data statistics, such as root hair density and number of root hairs.

We have included a classification with statistics to quantify root hair data of several figures. See also answer to point Nr 3.

9. In the abstract and introduction, the authors mentioned that cell wall formation id modulated by LRX/RALF/FER complex. But the related reference is missing.

The references listed on lane 76 do show data that changes in cell wall formation modulate the LRX/RALF/FER signaling module. Specifically, mutants affected in *FER* or *LRX3 LRX4 LRX5* are not able to properly respond to reduced pectin demethylesterification (Dünser et al., Ref Nr 15).

10. How to explain the root cell of apy7-1 elongate faster with a more alkali apoplastic pH?

The acid growth theory is a hypothesis and not a strict rule. There are studies showing that the theory does not always hold true. In fact, recent work has shown that the pH is lowest in a non-elongating zone of the root (Serre et al 2023, elife, Ref Nr 59). This point is now discussed (lanes 475-480).

11. The mechanism of apy7-1 affecting the cell wall components through RALF1/LRX/FER is not clear.
APY7 is likely a downstream component of processes influenced by the LRX/RALF/FER module. We found this interesting connection of ROS and APY7-GFP levels, in addition to an altered composition of Golgi-derived cell wall polysaccharides. This is discussed as a hypothesis on how RALF1/LRX1/FER signaling module can connect to APY7 (lanes 455-460, 494-498).

Minor
1. In Abstract, “LRX/RALF/FER” modules should be written in a same order. done
2. Line # 44: “resuling” is miswritten? done
3. Lines 123-125: The author should demonstrate the segregation ratio of F2 generation: how many lines were restored or not? done
4. Lines 149 and 374. “AYP7” and “AYP1” are miswritten? done
5. Error in Line 233, 1 uM RALF1. Line 257 1 μM RALF1. Should be unified. done
6. Line 264. An error in the unit of scale bar. done
7. Line 563: An error in “in the Leica SP5 microscope”. done
8. Line 568: Five biological replicates are not enough for HPTS assay.

This is an error in the Material and Method section that is corrected. The legend of the corresponding figure gave the correct number (10, already in the initial version).

9. Line 336: I suggest replace “cell wall structures” with “cell wall components”. done
10. The author compared the expression levels of APY7-GFP among different T2 lines through Western blot assay in Fig 1C. How to ensure that the loading amount is the same for each line? Please clarify it.

We have added now the PonceauS staining of this very membrane.

11. In Fig S2E, wild-type seedling should be added as a control under the same parameters to avoid the auto-flourescence. Besides, the value scale bar of this figure is missing. “ABCDE” font sizes are not uniformed.

The panels A-D have been adjusted. Autofluorescence is virtually non-existent in roots as this is mainly coming from chloroplasts. We had tested this in advance. It might become a problem at very low intensity of the GFP fluorescence. But here, the intensity is way higher.

12. In Fig4A, the position of apy7-1 root cell seems different from those of other samples.

Indeed, the representative picture of the apy7-1 mutant root is positioned slightly higher in the root and thus at the border to the elongation zone, which is the reason for the cells being slightly longer than in the other samples. Since it is the ratio of fluorescences that is most relevant here, the more alkaline pH is still observed in both apy7 alleles and hence consistent.

13. GFP intensity in Fig S2E and Fig S6 is too low to be convinced. One possible experiment is to investigate subcellular localization of APY7 and expression patterns in response to different concentrations of eATP.

The panel E in the suppl. Figure S2 is now augmented to better visualize the GFP fluorescence. The fluorescence is way above background and the patterning of the GFP fluorescence in speckles is reminicent of golgi-localization. This has also been reported by others, and we focused on other issues related to APY7 rather than the well-established sub-cellular localization. However, we have investigated the abundance of APY7-GFP upon ROS treatment since the production of ROS is influenced by the LRX/RALF/FER module. The findings on this analysis are described in the result section (lanes 324-339) and discussed (lanes 455-460).

14. The authors mentioned RALF 23 and RALF 33 in the method (Line 544). But no related data was presented in the manuscript. This has been removed.
15. It is counterintuitive that RALF1 can promote root growth in fer-4 in Fig 3C.

This is indeed counterintuitive, and we are not the only ones reporting this effect (e.g. Haruta et al Science, 2014). While it is potentially interesting, we believe this to be neglectable for our project, as the *fer-4* mutant was mainly used as a control for a mutant that fails to arrest root growth upon RALF1 treatment.

Why is there a huge difference in root lengths between Fig 3A and 3C, if the plants growth under similar conditions?

The reason for this discrepancy is that for the data of panel A, the seedlings were kept on agar plates in a vertical orientation while for panel C, the seedlings were germinated on agar plates and then transferred to liquid MS medium. Hence, the growth conditions are not identical. This is also mentioned in the legend, since it might indeed appear confusing at first.

16. Fig 4 shows an increase in cell wall pH values for the rol16 and apy7-1 mutants, indicating that APY7 affects the cell wall pH in root tissues. The authors should also present the apoplastic pH of fer5 rol16.

The point of this experiment is to show that the *apy7* alleles alter the pH of the extracellular matrix. To what extent they also affect the pH in combination with other mutants will eventually be interesting, but for would require the analysis of a number of mutants, and thus go beyond the scope of this manuscript. We feel that it is justified to not add additional mutant lines.

17. The authors analyzed the composition of the cell wall in seedlings using multiple monoclonal antibodies in Fig 7. Were the immunological efficiencies consistent for different samples?

Yes, the same antibody bound resulted in similar intensities in different experiments. The data are consistent. The sentence was adjusted to make this point clearer (lane 293-294).

18. There are many elements missed in the figures: statistical analysis in FigS1 A and Fig 5A; phenotypic image in Fig 3; scale bar in Fig S3C, Fig S4 and Fig 6; unit in Fig S6B; confocal image in Fig S6. Some of them are really important for the audience.

As requested, we have added data on SNP calls in WGS in the vicinity of the *APY7*/*ROL16* locus (new S1A Fig). Statistical analyses on the root hair formation defects were added to several figures.

In S6 Fig, the graph shows that the membrane fluorescence is clearly detectable, but there is not corresponding GFP signal at the membranes. We don’t have absolute values, but the GFP signal is not above background, since we had tested non-transformed plant material.

We believe that the phenotypic images for Fig 3A would not really provide additional information and that the graphical representation is a convenient way to oversee the data.

**Reviewer #2**: In this manuscript, the authors tested quite a few hypothesis, trying to decipher how LRX, FER, APY7 may involved in root hair formation. From my point of view, this manuscript is still preliminary, still need much more investigation.

Major points:
1. Fig 1 showed root hair defect in lrx1 mutant, the introduction of rol6 repressed root hair defect in lrx1 mutant, Fig 2 and S3A Fig showed similarity of lrx1/lrx2 to fer-4/fer-5, the introduction of rol6 repressed root hair defect in fer-5 mutant, but only partially repressed root hair defect in fer-4 background. This suggest that shorter root hairs in lrx1 may be similar to that in fer-5, but root hair defects (may be root hair burst) more severe than the shorter root hair in fer-4 are not repressed by rol6, which also suggest that root hair burst defect in fer-4 may not be correlated with APY7. The authors need to provide clear evidence showing different types of root hair defect in different mutants.

We are now providing a statistical analysis on the different types of root hairs observed.

2. Line 200-205, also in S3C Fig, the authors reported that rol16 boosted fer-4 defect in seed set. Together with their observation that rol6 only repressed the short root hair defect in fer-4, these data suggest that ROL6 may only function in ROS signaling pathway. The authors need to test whether lrx1 also have fertilization defect.

We have included data on ROS analyses: and we were not able to detect reproducible and consistent alterations by the *rol16* mutant. Interestingly, however, APY7-GFP fluorescence is differentially regulated with an increase in the tip but a decrease in the mature part of the root. Since we were not able to detect a change in ROS levels due to the *rol16* mutation, ROS has an impact on APY7 but not vice versa. These interesting data are included in the manuscript (lane 325-348 and new Fig 6) and are discussed (458-460).

The expression of *LRX1* is restricted to root hairs (Baumberger et al., Ref Nr. 23), which argues against a role of LRX1 in fertilization.

3. Line 215-270 (Fig 3, Fig 4) reported unrelated data, it is confusing to the readers why this is included. Why not directly describe Apyrase function starting from Line 272 (Fig 5)?

We have moved the paragraphs on cell wall composition/structure and ROS after the paragraph on extracellular pH. The paragraphs on the eATP sensing are now at the end of the manuscript (Fig 7).

4. Line 272-334 (Fig 5, Fig 6) reported the possible involvement of eATP and receptor proteins P2K1/DORN1 and P2K2. Again, this is not involved. Obviously, the gravitropic response caused by eATP is not correlated with root hair defect. Suggest to remove this data.

In view of the finding that *rol16* shows an altered response to eATP – as do the *dorn1* and *p2k2* mutants – we see the presented analysis as important. This reviewer is absolutely correct that neither DORN1 nor P2K2 are involved in the APY7-related effect, but this needed to be tested and was not obvious from the beginning. We believe that it is important to keep this data in the manuscript. It is also correct that we cannot claim a connection of APY7-influenced eATP sensitivity and root hair development, but also this needed to be analyzed. We believe that reporting the non-involvement of the eATP receptors is relevant.

5. Finally, based on staining of cell wall xylan and xyloglucan, the authors concluded APY7 influences cell wall structures, but they did not correlate this with cell wall defect. They also did not stain other root hair mutant with LM10 and LM15.

The change in immunolabelling indicates that the cell wall composition and/or structure is altered. The exact nature of these changes is not clear. This is an intrisic limitation of the immunolabelling approach. The data are now complemented with a cell-wall monosaccharide analysis presented in Fig 5 that shows that sugars found in polysaccharides synthesized in the golgi are reduced in *rol16*. This provides a better insight into the effects of the *rol16* mutation on cell wall formation.

Analyzing a series of cell wall mutants with LM10 and LM15 antibodies would be an option, but it would most likely not provide a better insight into the function of APY7. A possibility would be to combine *rol16* with many other cell wall mutants and look at the effect of *rol16* on their phenotypes. This will be an interesting future research direction but would go beyond the goal of this manuscript.

Minor points:
1. Pay attention to typo errors, such line:4-45：”resuling” in cells that are malformed, “to” small or big. “resuling” should be “resulting”, “to” should be “too” done
2. Line 123-125, “Backcrossing of the lrx1 rol16 mutant with lrx1 resulted in the F2 generation in a 3 : 1 segregation of lrx1 : wild type-like root hair formation, indicating that the rol16 mutation is recessive.” The conclusion from this experiment need to provide data and point to the corresponding supplementary data.

The initially determined number is now added (lane 128). This total number of seedlings (61) might not appear very high. But the segregation ratio of the *rol16* mutant is confirmed with 1000 seedlings (Suppl Figure S3B).

3. Line 196 showing in a rol16 mutant population segregating for fer-4 (rol16 -/- fer-4 +/-), however, only 12 out of 800 seedlings were homozygous for fer-4. But in S3B Fig, the sample name is written as rol16+/+; fer-4 +/-, should be corrected to rol16 -/- fer-4 +/-.

We apologize for this mistake that went unnoticed. It is now corrected.
